# Annual Dinner of the Erie Co. Medical Society

**Published:** 1858-02

**Authors:** 


### EDITORIAL DEPARTMENT.

*Annual Dinner of the Erie Co. Medical Society.*—The society met as usual on the second Tuesday in January, with quite a full attendance from the city and county; and after a session of more than ordinary interest, heightened in a great measure by the admirable address of its late president, Prof. Hamilton, and the oration of Dr. Newman, adjourned to meet again in the hospitable halls of the American Hotel of this city. At about four o'clock, between thirty and forty guests sat down to an excellent dinner, prepared by mine host of the American. Many of our readers were present, and those who were not, are sufficiently familiar with the effects of a good dinner and the physiology of the circulation of the bottle, to enable them to believe that every body was good natured, in good spirits, and making himself agreeable to himself and his neighbor.

The utmost geniality and good feeling prevailed, and a number of excellent and appropriate speeches were made in response to various sentiments, particularly the first speech by the president, and a characteristic and admirable response by the senior editor of the Journal, to a sentiment which those present will recollect, as well as speeches from the chairman of the dinner committee, the county members and others. All this contributed to enable the guests to pass four hours as pleasantly, and we think as profitably as possible.

Our profession has always been compelled to submit to jokes and insinuations concerning the differences which they say, must arise between doctors, but any one who could have looked in upon our society dinner, would have been compelled to acknowledge that there was one thing in which doctors did not disagree, but in which they were invariably unanimous, namely, the appreciation of a good dinner. Nothing brings men more closely together and goes farther to establish pleasant social relations, than a meeting of this kind, and if there be any truth in the imputation which is so frequently cast upon us, we can conceive of nothing which is better calculated to drown professional jealousy; and on the other hand, if there be no truth in it, a sufficient argument in favor of an annual society dinner, is the good time which we are all sure to have on the occasion. Older members of the society will recollect, (what we only know by tradition), the difficulty which was experienced in the establishment of a society dinner as a permanent institution; but now that it is established, we are confident that no one is blind to its advantages. May none of the number which met in 1858 be missing in 1859. F.

